# Knowledge translation and health technology reassessment: identifying synergy

**DOI:** 10.1186/s12913-018-3494-y

**Published:** 2018-08-30

**Authors:** Rosmin Esmail, Heather Hanson, Jayna Holroyd-Leduc, Daniel J. Niven, Fiona Clement

**Affiliations:** 1 Department of Community Health Sciences, Cumming School of Medicine, University of Calgary, 3D14A Teaching and Wellness Building, 3280 Hospital Drive NW, Calgary, Alberta T2N 4N1 Canada; 2 Alberta Health Services, Calgary, Alberta Canada; 3 O’Brien Institute for Public Health, University of Calgary, Calgary, Canada; 4 Department of Medicine, Cumming School of Medicine, University of Calgary, Calgary, Canada; 5 Hotchkiss Brain Institute, University of Calgary, Calgary, Canada; 6 Department of Critical Care Medicine, Cumming School of Medicine, University of Calgary, Calgary, Canada; 7 Health Technology Assessment Unit, University of Calgary, Calgary, Canada

**Keywords:** Health technology reassessment, Disinvestment, Knowledge translation, Implementation

## Abstract

**Background:**

Health Technology Reassessment (HTR) is an emerging field that shifts the focus from traditional methods of technology adoption to managing technology throughout its lifecycle. HTR is a mechanism to improve patient care and system efficiency through a reallocation of resources away from low-value care towards interventions and technologies that are high value. To achieve this, the outputs of HTR and its recommendations must be translated into practice. The evolving field of knowledge translation (KT) can provide guidance to improve the uptake of evidence-informed policies and recommendations resulting from the process of HTR. This paper argues how the theories, models and frameworks from KT could advance the HTR process.

**Discussion:**

First, common KT theories, models and frameworks are presented. Second, facilitators and barriers to KT within the context of HTR are summarized from the literature. Facilitators and barriers to KT include ensuring a solid research evidence-base for the technology under reassessment, assessing the climate and context, understanding the social an political context, initiating linkage and exchange, having a structured HTR Process, adequate resources, and understanding the roles of researchers, knowledge users, and stakeholders can enhance knowledge translation of HTR outputs. Third, three case examples at the individual (micro), organizational (meso), and policy (macro) levels are used to illustrate to describe how a KT theory, model or framework could be applied to a HTR project. These case studies show how selecting and applying KT theories, models and frameworks can facilitate the implementation of HTR recommendations.

**Conclusion:**

HTR and KT are synergistic processes that can be used to optimize technology use throughout its lifecycle. We argue that the application of KT theories, models and frameworks, and the assessment of barriers and facilitators to KT can facilitate translation of HTR recommendations into practice.

## Background

Health technology reassessment (HTR) is an emerging field that shifts the focus from traditional methods of technology adoption to managing technology throughout its lifecycle. HTR is defined as “a structured, evidence-based assessment of the clinical, social, ethical, and economic effects of a technology, currently used in the healthcare system, to inform optimal use of that technology in comparison to alternatives” [1–3]. Therefore, HTR is a mechanism to improve patient care and system efficiency through a reallocation of resources away from low-value care towards interventions and technologies of a higher value [1, 3–6].

To harness the potential power of HTR to reassess technologies currently in use, the outputs and recommendations must be translated into practice. This ‘translational’ step of the HTR process is felt to be the most difficult [3]. In a recent jurisdictional scan of HTR activities, Leggett et al. report that of the 16 organizations that had developed a HTR program, 54% had advisory capabilities with respect to the HTR decision, with less than 5% able to directly implement the decision [2]. Methods in knowledge translation (KT) could help to improve the uptake of evidence-informed policies and recommendations that result from a HTR process. In this paper we will argue, how the theories, models and frameworks from KT could advance HTR process. First, common KT theories, models and frameworks are presented. Second, facilitators and barriers to KT within the context of HTR are summarized from the literature. Third, three case studies are illustrated on how selecting and applying KT theories, models and frameworks can facilitate the implementation of HTR recommendations. To our knowledge there are no other publications on this topic and the arguments presented in this paper provide critical and much needed debate in this area.

### Clarifying the terminology of HTR

Before we begin to understand how KT theories, models and frameworks could advance HTR, it is important to understand the myriad of terms describing the field of HTR. The terminology surrounding HTR is vast; terms including waste, low-value, obsolescence, too much medicine, unnecessary care, overuse, disinvestment, de-adoption and de-implementation have all been used [3, 6]. In particular, terms such as disinvestment, de-adoption and de-implementation are used interchangeably with HTR. However, there are important differences among them. Disinvestment, is defined as the process of completely or partially withdrawing healthcare resources from currently funded areas that provide little benefit for their cost [7]. Disinvestment can lead to full or partial withdrawal of a technology, contractual variation, restriction, or substitution and employs financial disincentives [5, 8]. De-implementation is defined as the process where the use of low-value care is reduced or stopped on a structural basis in a planned process that uses a set of activities, which can include financial disincentives, but also uses other activities such as data feedback, education, and system interventions [8]. Finally, de-adoption is defined as the discontinuation or rejection of a clinical practice after it was previously adopted [9]. HTR is conceptualized as a process that offers a holistic approach to optimal technology use with disinvestment, de-implementation and de-adoption as possible outputs of the process [3].

### Conceptual model for HTR

Soril et al. recently proposed a conceptual model for HTR [3]. Briefly, the model has three phases: phase 1-technology selection (identification and prioritization); phase 2-decision (evidence synthesis and policy development); and phase 3-execution (policy implementation, monitoring and evaluation) [3].

Phase 1 involves the identification and prioritization of technologies. Technologies can be identified by clinicians, selected from pre-existing lists of low-value care, through horizon scanning or data driven mechanisms as benchmarking and practice variations [3, 7, 10]. Phase 2 consists of a broad evidence synthesis using methods primarily from health technology assessment (HTA) to determine the clinical, economic, ethical and social effects of the technology being reassessed. It also entails a comparison of alternative technologies [3]. Subsequently, based on the evidence synthesis and stakeholder input, a policy or practice recommendation is generated. At the end of this phase, there are four potential outputs: an increase in utilization or adoption, a decrease in utilization, no change in utilization, or withdrawing the technology completely [3]. Phase 3 entails the implementation of the policy or practice recommendation followed by monitoring and evaluation. At the end of phase 3, the ultimate outcomes of the HTR process are assessed by determining if the change was achieved or not, or remained at status quo. Note, a distinction is being made between the outputs or activities that are the result of the process in phase 2 and the outcomes or the impact that has occurred as a result of implementing the activities in phase 3.

The model proposed by Soril et al. also has two cross-cutting foundational elements that span all three phases: meaningful stakeholder engagement and ongoing knowledge exchange and utilization. Stakeholder engagement involves individuals or groups throughout the HTR process who may be impacted by the decision. Stakeholders can include physicians, nurses, other clinicians, hospital managers, decision makers, government and the public [3]. Continuous knowledge exchange is important throughout the HTR process and involves stakeholders in the selection, prioritization, and identification of reassessment topics, the development of research questions, knowledge generation, and interpretation of findings. Both foundational elements are necessary for the successful implementation of HTR recommendations [3]. This paper addresses the question how do the outputs in phase 2 of this HTR model translate to the outcomes in phase 3 and can KT play a role here?

### Relevant knowledge translation theories, models and frameworks

KT is defined as a dynamic and iterative process that includes the synthesis, dissemination, exchange and ethically-sound application of knowledge to improve the health of populations, provide more effective health services and products, and strengthen the healthcare system [11]. Thus, KT has emerged as a field of medicine that bridges the gap between “what we should be doing” and “what we are actually doing” in practice [12, 13].

There are several KT theories, models, and frameworks that have been summarized in various narrative and scoping reviews. [11, 14–17]. Depending on the criteria used, there are between 40 to 60 KT models currently described in the literature [11, 14–17]. The most frequently cited models and frameworks are: RE-AIM (reach, efficacy, adoption, implementation and maintenance), translation research continuum or ‘T’ models also known as bench to bedside models, the Knowledge-to-Action (KTA) framework, the promoting action on research implementation in health services (PARiHS) framework, the evidence-based public health models, stages of progression (rocket model), interactive systems framework for dissemination and implementation, and the UK medical research council framework [17]. These KT theories, models, and frameworks all acknowledge the gap between research and its application in practice and policy. They also acknowledge the challenge that exists in bridging this gap. Many provide a process through a description of key components or illustrate diagrammatically how evidence and evidence-based interventions can be applied in practice or policy. However, these KT theories, models, and frameworks often lack details of how to scale up interventions that are shown to be successful, none incorporate cost or cost-effectiveness as a factor in determining if an intervention will be adopted widely and many of these models have yet to be tested [17].

Knowledge translation science theories, models and frameworks can be classified using five categories of theoretical approaches (Table 1) [16]. These categories classify KT theories, models, and frameworks based on their purpose and approach. The taxonomy provides a concise way to frame and select from the myriad of approaches that are available to the researcher based on the situation. Further, there may also be merit in combining approaches to understand and drive the implementation process. Thus, it may be entirely acceptable to use more than one theory, model or framework. Moreover, the use of these theories, models and frameworks will depend on the knowledge users themselves (clinicians versus policy makers); their goals, and the level at which the change is being implemented. These levels can be at the individual (micro level), organizational (meso level), or policy (macro level).

**Table 1:** Five Categories of Theoretical Approaches to Knowledge Translation

Category	Definition	Exemplar Model
Process models	Specify steps in the process of translating research into practice	Ottawa model on research use [28]-six key elements: evidence-based innovation; potential adopters; the practice environment; implementation of interventions; adoption of the innovation; and outcomes resulting from implementation of the innovation. Each element is assessed, monitored and evaluated before, during and after the decision is made to implement an innovation
Determinant frameworks	Classes or domains of determinants that are hypothesized or have been found to influence implementation outcomes	Promoting action on research implementation in health services (PARiHS) [38]-three determinants are evaluated context, evidence and facilitation from low to high to determine how they can impact change
Classic theories	Describe how change occurs without ambitions to actually carry out the change	Rogers’ diffusion of innovation theory [9]-theory that explains how a change occurs
Implementation theories	Developed and adapted by researchers for potential use in implementation science to achieve enhanced understanding and explanation of certain aspects of implementation	COM-B – capability, opportunity and motivation framework [16]-these 3 factors explain whether a behavior change will occur
Evaluation frameworks	Provide a structure for evaluating implementation endeavors	RE-AIM [39]-reach, efficacy, adoption, implementation and maintenance are factors to evaluate the extent to which implementation has occurred

**Fig. 1:**
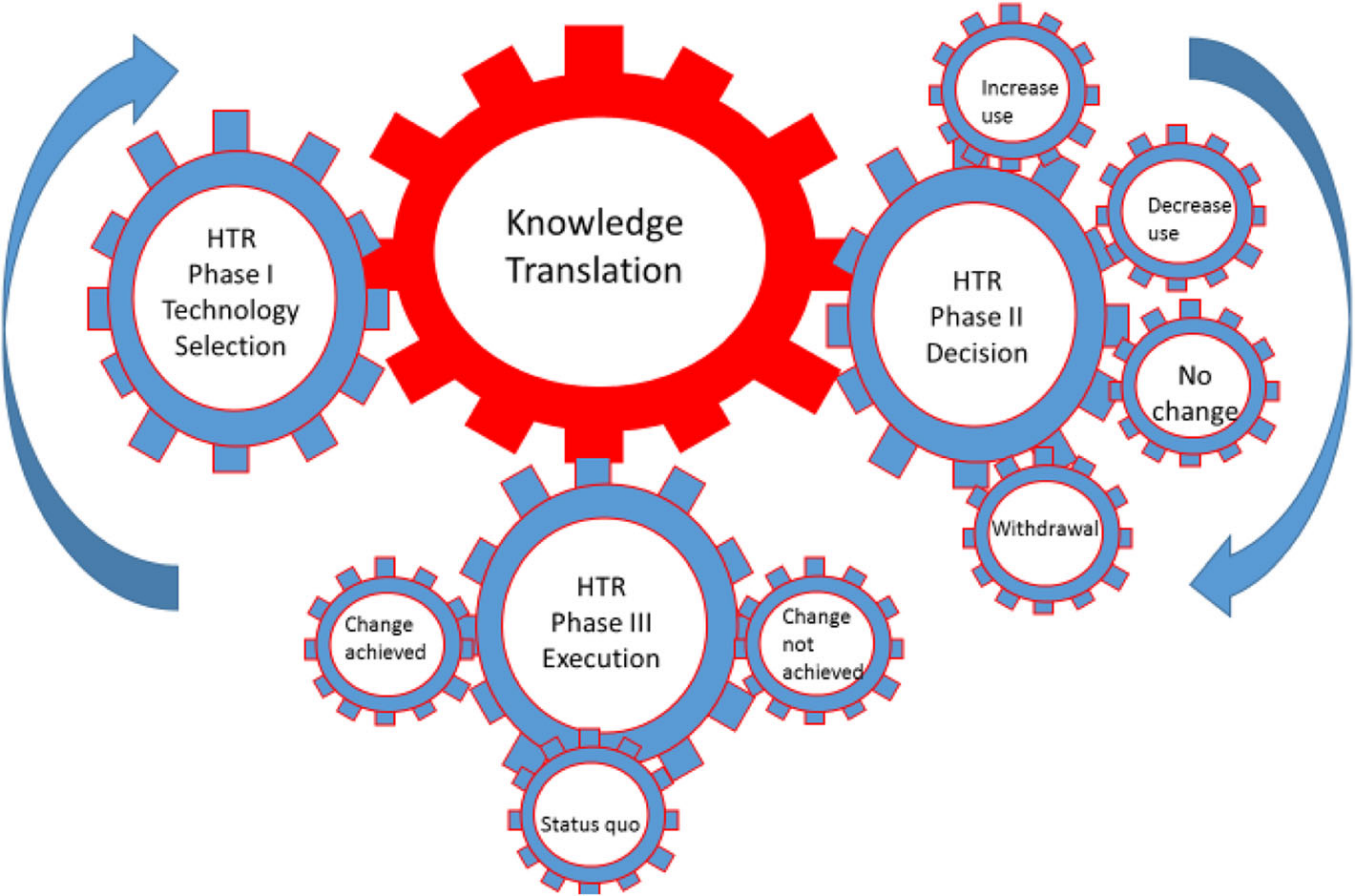
Linkage between Knowledge Translation and Health Technology Reassessment

The field of KT and its application to HTR is currently untouched and underutilized. We argue that KT approaches be used in the HTR process to bridge the gap between the generation of recommendations regarding technology use and their implementation. We posit that the methods in KT are essential to the success of HTR. KT’s role within the HTR context is central, driving the entire process as described in Fig. 1. Subsequently, KT models, theories and frameworks when applied to HTR could be used to move knowledge into practice.

### Barriers and facilitators to knowledge translation within the context of HTR

Prior to applying KT approaches to HTR, understanding the barriers and facilitators to KT within the context of HTR is important initial step. Assessment of these barriers and facilitators need to become common to the practice of HTR. Yet, making this happen remains elusive. To frame this discussion, we have used the World Health Organization’s classification of barriers and facilitators is used (Table 2) [11].

**Table 2:** Barriers and Facilitators to HTR, Disinvestment, De-implementation, and De-adoption (*Barriers specific to implementation of HTR recommendations)

Modified WHO Classification [11]	Sub-Categories	Barriers	Facilitators
Climate and Context Individual’s negative attitudes, overall sense of political will, and openness to research	Health Care Providers	Physicians are reluctant to dismiss outmoded devices and procedures [4] Lack of incentives to decrease or remove technologies [22]	Use of clinical champions [5] Involve clinicians to increase buy-in [2] Address perceived net benefit to patients [8]
	Patients/Public	Removal of technologies and procedures may cause concern for health professionals and patients who will view the exercise as a reduction of available health services [22]	Shared dialogue [21]
	Political/Social/Decision makers	Political and social barriers/push back [2, 19, 22] Absence of political drive [21] Lack of support from decision makers [20]* Lack of collaboration [19, 21]	Political support [5, 21] Government interest [2] Local/national relationships [5] Policy regulations and restrictions [3, 8] Encouragement of political discussion and raising awareness before and during implementation [20]*
Linkage and Exchange Underlying linkage and exchange between researchers and knowledge users, policy makers and stakeholders		Lack of a well-planned implementation strategy that involves all stakeholders and is aligned with the initial goal of the program [20]* Absence of strong leadership [20]* Concept of low-value care not understood [24] Cost savings viewed as unfavourable [24]	Broad and early stakeholder engagement [2, 20] Meaningful stakeholder engagement and on-going knowledge exchange [3] A dissemination strategy tailored to target groups [20]* Consideration of local contexts [20]* Use of clinical champions [5] Address perceived net benefit to patients [8] Public representatives involvement in the process to increase knowledge of the HTR process [2] Shared dialogue [21] Do not frame as ‘waste’ but focus more on ‘harm’ and staged testing and treatment [25] Coordination/collaboration/professional understanding [5, 21]
Research Evidence, a Structured HTR Process, and Resources Timeliness, relevance and local applicability of research		Lack of methods to identify technologies with uncertain cost-effectiveness [19] Lack of understanding and expertise of HTR [2, 19, 21] Lack of approaches to conduct a HTR that are transparent [21] Lack of relevant evidence of the technology itself [18, 19]	A structured evidence-based process that includes transparent methods for identification, prioritization, and assessment of ineffective health technologies [20]* Good evidence base for identification and recommendations [20]* Mitigate with clear identification and prioritization criteria [7, 10] Additional human and financial resources for sustainable implementation [20]*
Role of Researchers and HTR The role of researchers to facilitate the transfer of research which includes views of their own role, communication skills, and packaging of the research results		Researchers may not understand their role Financial resources for HTR [20] Lack of resources and human resources to support HTR [19, 20]* Large investment in work and time required for HTR [19, 21] Difficulty in communicating with a variety of audiences and public perceptions [2, 5]	Capacity building in KT and change management [1] Understanding KT theories, models and frameworks and use of effective and multifaceted KT interventions [1] Development of a KT strategy to ensure uptake of HTR recommendations ([20, 22]
Role of Stakeholders, Knowledge Users and the Health System in HTR Skills and expertise		Lack of skills [2] Lack of resources and human resources to support HTR [19–21]* Absence of strong leadership [20]*	Raising awareness and leadership at all levels [1, 20] Use of change agents or knowledge brokers [12] Decision makers need to understand the HTR process and provide support

HTR involves the evidence synthesis of technologies that are currently used in the system. However, for some technologies, there may be a lack of evidence regarding their effectiveness and/or cost-effectiveness [18, 19]. A good evidence base for the identification of potential technologies is essential [3, 20]. Alternatively, mechanisms to obtain this evidence through ‘evidence generation activities’ could be undertaken [7, 18]. Additionally, there may be a lack of understanding and expertise of HTR, a lack of methods to identify and prioritize technologies, and a lack of transparent approaches to conduct a HTR [2, 18, 19]. Thus, a transparent, structured, evidence-based process for the reassessment of technologies with clear identification and prioritization criteria is required [3, 7, 10, 20]. In addition, the consideration of time, human and financial resources is needed to sustain a viable HTR process [19–21].

Climate and context in which the proposed change is to be implemented manifests itself in HTR through barriers related to a health care provider’s attitudes towards the change, the social, and political context. Although providers may have the best intentions to adhere to HTR recommendations, intentions do not automatically equate to behaviour change, particularly if they involve the decreased use or elimination of a technology. Providers may be resistant or reluctant to dismiss technologies [4]. There may be a lack of incentives to decrease or remove technologies from the system [22]. Psychologically, it is harder to stop something in practice [8]. Additionally, providers could succumb to pressure from the patient in providing unnecessary tests or treatments. Facilitators to overcome these barriers include the use of KT strategies such as clinical champions to increase buy-in, and providing knowledge of the net benefit to patients if the technology is removed or decreased [2, 5, 8]. Knowledge translation and implementation of HTR recommendations could also be motivated by explicitly stating that the intention is to improve efficiency, patient safety and patient outcomes, rather than cost savings [21]. A shared dialogue between providers and patients may also be required [21]. Lastly, as part of the KT plan development, providers could be asked from their perspective, what their beliefs, knowledge, skills, experiences, attitudes and motives are with respect to a technology prior to undertaking a HTR [23].

Understanding the social and political context within which a HTR is being undertaken is also key [2, 19–22]. There could be public pressure or the need for government to follow another jurisdiction’s decision on a particular technology. Political support, collaboration with government, and building relationships are important mitigating factors [2, 5, 20, 21]. In addition, policy regulations and restrictions in funding could be the policy change levers used to implement HTR recommendations [3, 8].

A lack of appropriate linkage and exchange with all stakeholders (providers, patients, policy makers and other stakeholders) is another barrier to HTR. Meaningful stakeholder engagement and on-going knowledge exchange have been emphasized as cross-cutting foundational elements of the HTR model [3]. Broad and early stakeholder engagement, trusting and respectful relationships, and face-to-face interactions are facilitators to ensure ongoing linkage and exchange. From a provider perspective, strong leadership, the use of clinical champions, and understanding their perceptions is key [2, 5, 20]. From the public/patient perspective, the concept of HTR or ‘low-value care’ may not be well understood [24]. Moreover, cost savings as an objective of HTR is also viewed as unfavorable [24]. Hence, having public representatives involved in the process to increase their knowledge and understanding, a shared dialogue amongst patients and providers, and framing low-value practices not as a ‘waste’ resulting from financial costs associated with unnecessary tests and treatments but as ‘harm’, including physical, emotional and financial harms, are strategies to support linkage and exchange [2, 20, 21, 24, 25]. From a policy maker perspective, encouragement of political discussion and raising awareness before and during implementation [20], coordination, collaboration, and professional understanding would all be important facilitators for ongoing linkage and exchange [5, 21].

Researchers involved in HTR may not understand their role within the process. Furthermore, these individuals may lack the necessary skills to communicate and interact with stakeholders. They may also lack knowledge in the use of KT theories, models, and frameworks or skills to facilitate implementation. Capacity building of these individuals in the fields of KT and change management is one way to address this barrier [1]. Additionally, understanding KT theories, models, and frameworks and the use of effective and multifaceted KT interventions to implement HTR policy or practice recommendations is needed [1]. Researchers, with stakeholder input, could develop a well-planned overall KT strategy that is aligned with the initial goal of the HTR, uses KT approaches, and is tailored to stakeholders to ensure the uptake of HTR recommendations [20, 22].

Stakeholders play a key role in the uptake of HTR evidence. Barriers such as lack of skills and the system’s poor capacity to access HTR exist [2, 19, 21]. There may also be the absence of strong leadership to undertake a HTR or implement its recommendations within an organization [20]. These barriers can be mitigated through capacity building of stakeholders, raising awareness of HTR, and leadership support at all levels [1, 20]. The use of change agents or knowledge brokers to bridge the gap between those involved in the HTR process and decision makers could also enhance HTR efforts [12]. Knowledge translation and implementation within the health system may not be the problem, but rather it is ensuring that operationalization of the recommendations occurs within a functional decision-making infrastructure [26]. Decision-makers need to understand the HTR process and provide the support and mechanisms to implement its recommendations within the health care system.

As described in Table 2 there are several barriers and facilitators of KT within the context of HTR. Those that are significant to the implementation of HTR recommendations have been identified in the literature [20]. Within the context of HTR, our position is that these are all critical and important to address if the recommendations from the HTR are to be implemented in practice.

### Application of KT to HTR - Three examples

To better understand how KT theories, models and frameworks could fit within the context of the HTR, we propose three examples to illustrate the process at each of the three different levels within the healthcare system (individual-micro, organizational-meso, and policy-macro levels).

### Individual level - Micro system example

The overuse of antipsychotics to treat the behaviour and psychological symptoms of dementia in long-term care settings is a major concern [27]. With the assistance of the University of Calgary’s Health Technology Assessment unit, Alberta Health initiated a HTR [27]. Subsequently, a practice recommendation was generated that results in the appropriate use of antipsychotics in long-term care settings. At this micro level, a process KT model could be applied to change this practice at a long-term care setting. The Ottawa model for research use could be used to assess, monitor and evaluate potential innovations to influence the prescribing patterns of providers. This model is useful to facilitate knowledge translation at the local context and its focus is on the continuity of care intervention which by its very nature is complex bridging sectors, settings and provider groups [28]. The model has six key elements: evidence-based innovation; potential adopters; the practice environment; implementation of interventions; adoption of the innovation; and outcomes resulting from implementation of the innovation. KT interventions may include the use of clinical champions, clinical practice guidelines, staff education, monthly inter-professional medication reviews, clinician reminders, care plan reviews, involving family, and audit and feedback [5, 6, 18, 29–31]. In addition to this model, a combination of KT theories, models, and frameworks may be beneficial to assess barriers and facilitators to prescribing practices of these medications [16]. In this example, prior to selecting interventions, interrogating which behaviour change interventions are effective may be useful. In fact the authors of the OMRU model suggest just that through the concept of ‘theoretical pluralism’ where other theories, models, frameworks could be applied to components of the model [28]. The theoretical domains framework is based on a review of 33 psychological theories and constructs to determine those most relevant to behaviour change [16, 32, 33]. Fourteen domains of behaviour change, such as knowledge, skills, intentions, goals, etc. have been mapped to 137 behaviour change techniques. So, if knowledge was identified as a behaviour domain, education and information are behaviour change techniques that could be used to influence the prescribing patterns of providers [33].

### Organizational level - Meso system example

Primary care networks in Alberta embarked on a HTR to reassess imaging for low back pain [34]. A practice recommendation was generated to decrease imaging for patients with low back pain at all primary care practices in the province [25]. At this meso level, a modified version of the KTA framework, the synthesis model for the process of de-adoption, could be applied [6]. To our knowledge this is the first de-adoption model that has been created and the model may be useful to apply in the de-adoption of imaging for low back pain. This model was developed through the identification of themes common to frameworks from a scoping review on de-adoption and modification of the KTA framework. The de-adoption model focuses on the identification and prioritization of low-value clinical practices with stakeholder engagement and follows the action cycle of the KTA framework to move these low-value practices into action or to reduce their use [6]. It is intuitive and easy to understand, however is has yet to be tested in practice. De-adoption change interventions could include information leaflets for patients, provider education, clinical decision support, a reduction toolkit, audit and feedback, and low back pain guideline changes for family physicians. These interventions would be tested, implemented, monitored and evaluated as part of the action phases of the KTA framework [6].

### Policy-level – Macro system example

The Australian government initiated a review of the policy on in-vitro fertilization [35]. At present, Australians are eligible for this subsidy regardless of age and prior treatment. Through the Medical Services Advisory Committee that makes recommendation to the Health Minister on which medical services should be publicly funded, a HTR was undertaken with key stakeholders. Subsequently, based on deliberative engagements with three key stakeholder groups, a policy recommendation to only publicly fund in-vitro fertilization for women between the ages of 21 and 45 was generated [35]. At the macro level, the implementation of this recommendation may require a different KT model to be applied. The Linking Research to Action framework (RTA) is selected as it assesses efforts and provides activities to consider to inform health policy decisions [11]. At the policy level, there are other factors that need to be addressed that may be different from the micro and meso levels. This model considers these factors through four elements: climate for research use, production of research and appropriate synthesis for policy makers, linking research to action strategies-push/pull/exchange efforts, and evaluation. The RTA framework could be adapted to assess the climate, including ethical considerations and perspectives of providers/patients; determine the level of research evidence required for policy makers; ascertain strategies to implement the policy, such as funding restrictions, and evaluate the policy post-implementation.

Thus, we contend that the selection of a KT theory, model or framework within the context of HTR will depend on the HTR outputs and the levels at which knowledge needs to be translated (i.e. individual, organizational, policy). These three examples provide suggestions of potential KT theories, models, and frameworks that could be used. None have been applied within the context of HTR, which has been identified as an area for further research [3, 6, 22, 36]. Moreover, KT theories, models and frameworks may need to be adapted and utilized early on within the HTR process [6, 23, 37].

## Conclusions

This paper demonstrates the synergy between KT and HTR and the role KT can play in the translation of HTR recommendations. We posit that KT theories, models and frameworks in the implementation of HTR recommendations can be applied to facilitate implementation of HTR outputs at the micro, meso and macro levels. That is, in the HTR conceptual model, KT could facilitate the translation of outputs from phase 2 to the outcomes in phase 3. Research regarding which KT theories, models, and frameworks may be best suited alongside the HTR process is critically needed. Overall, HTR and KT are complimentary processes that could be used to optimize technology use throughout its lifecycle. Let the dialogue begin!

## Abbreviations

HTA: Health technology assessment
HTR: Health technology reassessment
KT: Knowledge translation
KTA: Knowledge-to-Action (KTA) framework
PARiHS: Promoting action on research implementation in health services framework
RTA: Linking research to action framework
